# Influence of lifestyle and genetic variants in the *aldo-keto reductase 1C3* rs12529 polymorphism in high-risk prostate cancer detection variability assessed between US and New Zealand cohorts

**DOI:** 10.1371/journal.pone.0199122

**Published:** 2018-06-19

**Authors:** Nishi Karunasinghe, Stefan Ambs, Alice Wang, Wei Tang, Shuotun Zhu, Tiffany H. Dorsey, Megan Goudie, Jonathan G. Masters, Lynnette R. Ferguson

**Affiliations:** 1 Auckland Cancer Society Research Centre (ACSRC), Faculty of Medical and Health Sciences (FM&HS), The University of Auckland, Auckland, New Zealand; 2 Laboratory of Human Carcinogenesis, National Cancer Institute/NIH, 37 Convent Drive Bethesda, MD, United States of America; 3 Urology Department, Auckland City Hospital, Auckland, New Zealand; 4 Discipline of Nutrition and Dietetics, FM&HS, The University of Auckland, Auckland, New Zealand; Ohio State University Wexner Medical Center, UNITED STATES

## Abstract

**Introduction:**

The prostate-specific antigen (PSA) based prostate cancer (PC) screening is currently being debated. The current assessment is to understand the variability of detecting high-risk PC in a NZ cohort in comparison to a US cohort with better PSA screening facilities. Aldo-keto reductase 1C3 (AKR1C3) is known for multiple functions with a potential to regulate subsequent PSA levels. Therefore, we wish to understand the influence of tobacco smoking and the AKR1C3 rs12529 gene polymorphism in this variability.

**Method:**

NZ cohort (n = 376) consisted of 94% Caucasians while the US cohort consisted of African Americans (AA), n = 202, and European Americans (EA), n = 232. PSA level, PC grade and stage at diagnosis were collected from hospital databases for assigning high-risk PC status. Tobacco smoking status and the AKR1C3 rs12529 SNP genotype were considered as confounding variables. Variation of the cumulative % high-risk PC (outcome variable) with increasing PSA intervals (exposure factor) was compared between the cohorts using the Kolmogorov-Smirnov test. Comparisons were carried out with and without stratifications made using confounding variables.

**Results:**

NZ cohort has been diagnosed at a significantly higher mean age (66.67± (8.08) y) compared to both AA (62.65±8.17y) and EA (64.83+8.56y); median PSA (NZ 8.90ng/ml compared to AA 6.86ng/ml and EA 5.80ng/ml); and Gleason sum (NZ (7) compared EA (6)) (p<0.05). The cumulative % high-risk PC detection shows NZ cohort with a significantly lower diagnosis rates at PSA levels between >6 - <10ng/ml compared to both US groups (p<0.05). These were further compounded significantly by smoking status and genetics.

**Conclusions:**

High-risk PCs recorded at higher PSA levels in NZ could be due to factors including lower levels of PSA screening and subsequent specialist referrals for biopsies. These consequences could be pronounced among NZ ever smokers carrying the *AKR1C3* rs12529 G alleles making them a group that requires increased PSA screening attention.

## Introduction

Since PSA was considered a marker for prostate cancer (PC) diagnosis by the US Food and Drug Administration in 1994 [1], US health services had a dedicated PC screening system until the year 2008. Since 2008, PC screening with PSA was restricted to those <75y based on the US Preventive Services Task Force (USPSTF) recommendations [2]. In 2012 USPSTF increased restrictions and placed a D grade recommendation for discouraging PC screening with PSA. This was largely based on review of findings on benefits and harms of PSA screening from two large scale studies (the European Randomized Study of Screening for Prostate Cancer (ERSPC) [3] and the US Prostate, Lung, Colorectal and Ovarian Cancer Screening Trial (PLCO)) [4] as reviewed by Fleshner et al [5]. The ERSPC study has shown a reduction of mortality from PC while the PLCO study has shown no benefit [3, 4]. Meanwhile, a 10y follow-up mortality rate has been compared between a group of men from UK attending to a single invited PSA screening and a control group receiving only standard practice without screening [6]. These authors report that there was no difference between 10y follow-up mortality rates of the two groups except for an increase of low-risk PCs (Gleason grade <6) in the screening group. However these authors note that the 10y follow-up time may not have reached the 12y lead time for PC development as recorded for patients from UK [7]. According to an analysis of data from the National Health Interview Survey (NHIS) in US, 34% men have been PSA screened between 2000–2005, 36% in 2010 and 31% in 2013 [2]. In NZ there was no dedicated PC screening system in place and PSA testing or digital rectal examination or both were undertaken at varying levels (7–41%) by primary care physicians [8]. Obertova et al reports that 28.7% and 20.7% of men registered with medical practices in urban and rural areas respectively in the Midlands region in NZ were PSA tested in 2010 [9]. These authors further indicate a gap in being referred to a specialist or getting biopsied for men with elevated PSA levels. The proportion of men with elevated PSA levels that received subsequent biopsies in the Midland region in NZ in 2010 were 22.6% and 29.8% respectively among urban and rural centres [9]. These authors have noted a significant difference in the proportions of biopsies recorded from urban and rural settings when data were stratified by an age cut off of 70y. These biopsy rates in men with elevated PSA levels were much lower than those recorded for a US centre during pre- (44.3%) and post- (45.5%) USPSTF PSA recommendations 2012 [10]. In the year 2013, recommendations were made by the New Zealand Prostate Cancer Taskforce (NZPCTF) with regards to the diagnosis and management of PC in NZ Men [11]. According to the NZPCTF there was not sufficient evidence to support organised national PSA screening to outweigh the benefits over the harms of over-diagnosis and over- treatment of PC in NZ.

Welch and Black have reviewed literature on the proportion of men unknowingly carried PC up to the time of death by causes other than PC [12]. In this analysis, these authors have estimated a PC reservoir of 30–70% among men older than 60y. This means that men having indolent PC could have a near normal lifespan. Therefore it is mostly those with high-risk disease that will benefit from screening, diagnosis and management towards survival benefits.

We have previously recorded that tobacco smoking is associated with an increase in serum PSA levels in a mixed cohort of men consisting of healthy individuals as well as those with urology disease including PC [13].There is a notable involvement of the *aldo-keto reductase 1C* enzymes in the metabolic activation of chemical carcinogens such as those derived from tobacco constituents consisting of polycyclic aromatic hydrocarbons [14, 15]. Meanwhile AKR1C3 is also known to catalyse extra-testicular androgen synthesis [16]. Therefore, in the event of an increased burden of tobacco metabolism that requires AKR1C3 intervention extra-testicular androgen production could be compromised, subsequently leading to lower PSA levels. We have assessed the association of four androgen pathway related genetic polymorphisms (*Steroid 5 Alpha-Reductase 2* (*SRD5A2*) rs632148, *Cytochrome P450 Family 17 Subfamily A Member 1* (*CYP17A1*) rs743572, *AKR1C3* rs12529 and *Microseminoprotein Beta* (*MSMB*) associated SNP rs10993994) with PC risk and associated factors in a cohort of men from Auckland, NZ [13]. Out of these SNPs the *AKR1C3* rs12529 *G* allele in particular showed a negative association with serum PSA level when interacting with age, alcohol consumption, BMI, and smoking status in PC patients when compared to healthy controls [13]. This could imply that men carrying the variant allele *G* of the *AKR1C3* rs12529 genetic polymorphism could get under-detected for significant PC when interacting with factors including lifestyle. This also means that the AKR1C3 rs12529 C allele will have a positive association with serum PSA level and even could lead to over-diagnosis and over-treatment for non-significant PCs. We have also recorded that the *AKR1C3* rs12529 G allele is positively associated with androgen deprivation therapy related side effects [17]. We have reviewed various aspects of the influence of AKR1C3 in PC [18] and are of the view that the *AKR1C3* rs12529 genetic polymorphism could be a candidate confounding variable for patient stratification in the current analysis along with tobacco smoking status.

The impacts of such lifestyle and genetic variability on diagnosing significant PC could be understood better if compared between different PSA screening regimes such as in NZ and US.

## Methods

### Patient recruitment and data collection

The NZ patient cohort considered here was from the ‘Genomic studies on Prostate Cancer’ carried out at the University of Auckland in collaboration with the Urology Department, Auckland City hospital. The recruitment process involved, inviting men of any ethnicity with positive biopsies for PC from the Auckland Regional Urology Registry (Auckland, Middlemore, and North Shore hospitals), NZ. Recruitment was carried out at the Green Lane Outpatient’s Clinic, in Green Lane, the Manukau Super Clinic in Manurewa, and the North Shore hospital in Takapuna. Recruitment was restricted to men between 45-90y attending the clinics for follow up before or after the surgery, hormonal or radiation therapy, chemotherapy, or those on active surveillance or watchful waiting. Patient recruitment in NZ was initiated in October 2006 and ended in December 2013. Initially NZ patients were recruited within one year of diagnosis, if they had not undergone any treatment for PC. In 2008, the criterion was relaxed to include all patients with malignancies but within one year of diagnosis. In September 2010, the time frame for recruitment was altogether removed. A total of 408 men were recruited from NZ to the study. Clinical and pathology records of patients were evaluated at the hospital databases to collect age, PSA level, disease stage [tumor-node-metastasis (TNM)] and Gleason grade at diagnosis. Disease stage/prognostic grouping followed the criteria defined by the 7^th^ edition of the American Joint Committee on Cancer (AJCC) abbreviated as I, IIA,IIB,III and IV. D’Amico et al retrospectively monitored a PC patient cohort undergone radical prostatectomy, and radiation implant with or without neoadjuvant androgen deprivation therapy towards an outcome measure of PSA failure [19]. Based on this outcome measures, these authors were the first to stratify high-risk PC patient category as those having a clinical tumour stage ≥T2C or a PSA level of >20ng/ml or a Gleason grade of ≥8. In the current analysis high-risk PC was stratified based on a cancer stage of ≥T2C or PSA≥20ng/ml or a Gleason grade of ≥8 using a recently reported criterion [20, 21]. This high-risk PC stratification fitted well with a cut-off at stage/prognostic group IIB defined by the AJCC. Ethnicity and lifestyle data including tobacco smoking habits were collected using a self-administered questionnaire. Tobacco smoking lifestyle (current/lifetime) was recorded as current or former (considered together as ever smoker) or never. There was no threshold set for identifying never smokers. The NZ cohort consisted of 94% of men with European ancestry and 5.2% of Pacific, East Asian, Indian and Middle Eastern ancestry while for 0.8% ethnicity was not recorded. All these patients were considered under the NZ cohort.

The US patient groups were from the NCI-Maryland Prostate Cancer Case-Control Study. The study was initiated to test the primary hypothesis that environmental exposures and ancestry-related factors contribute to the excessive PC burden among African-American men when compared to European-American men. Therefore, other ethnicities were not included in this recruitment [22]. The study was initiated in 2005 and recruitment ended in 2015. The catchment area for the cases included the greater Baltimore area, Maryland, Washington DC, and few neighbourring counties in Pennsylvania, Delaware, and Virginia, but the majority of the recruited men resided in four Maryland counties: Anne Arundel, Baltimore City, Baltimore County, and Howard. These areas have a large African-American population. Patients were recruited at two hospitals, the Baltimore Veterans Affairs Medical Center and the University of Maryland Medical Center, that treat patients who are mainly of African-American and European-American race/ethnic background. Other minorities are uncommonly seen as patients in these two hospitals. Recruitment was restricted to those with a PC diagnosis within the last two years prior to study initiation. Other inclusion criteria required that the men were 40 to 90 years old, were born in the US, and spoke English well enough to be interviewed. Severely ill men or men residing in an institution were not eligible for the study. These men self-reported to be either AA or EA at an interviewer administered questionnaire completion and signed an informed consent to participate in the study. The survey also evaluated family health and lifestyle factors that included tobacco smoking habits. Tobacco smoking lifestyle (current/lifetime) was recorded as current or former (considered together as ever smoker) or never. There was no threshold set for identifying never smokers. Of the 976 cases that were recruited into the study, 489 were AA and 487 were EA. Patient clinical information (age, PSA level, TNM stage and Gleason grade at diagnosis) was collected from pathology reports and medical records. Disease stage/prognostic grouping and high-risk classification followed the criteria as mentioned before.

### Ethics approval and consent to participate

Patient recruitment from NZ was carried out with informed written consent under the Northern B (former Northern Y) ethics approval NTY/05/06/037 during the period 10/10/2006 to 02/12/2013. Patient recruitment from US too was carried out with informed written consent after approval by the NCI (protocol # 05-C-N021) and the University of Maryland (protocol #0298229) Institutional Review Boards.

### SNP genotyping

At recruitment, patients provided a blood sample. DNA from the NZ cohort was extracted from EDTA bloods using a QIAamp genomic DNA kit (Qiagen, Hilden, Germany) following the manufacturers’ protocol with the aid of a fully automated QIAcube (Qiagen, Hilden, Germany). For the US groups blood was spun at 850g for 10min at 4°C and plasma was aspirated. Buffy coat was removed from the remaining red blood cell pellet and washed once in phosphate buffered saline pH 7.4. DNA was extracted from the buffy coats using the DNeasy blood and tissue kit from Qiagen (Qiagen Hilden, Germany) following manufacturers’ instructions. SNP genotyping for the *AKR1C3* rs12529 was carried out using either the Sequenom MassArray system [23–25] (multiplexed with others SNPs) according to manufacturer’s instructions (Sequenom, San Diego, CA, USA) as described in Ferguson et al. [26] or a TaqMan® SNP Genotyping Assay from Applied Biosystem (AB) using AB 7900 Real-Time PCR system for the NZ cohort [17, 26]. For the US cohorts the same TaqMan® SNP genotyping procedure was followed on an AB 7500 Real-Time PCR system. The TaqMan® assay uses allele specific, dual-labelled hybridization probes from a predesigned assay on demand (C__8723970_1). A total of 408 patients from NZ and 474 from US were genotyped for the *AKR1C3* rs12529 SNP and results received for a total of 389, 207 and 249 respectively for NZ, AA and EA patients.

### Data stratification

PC patient data carrying race/ethnicity, smoking status, age, PSA level and Gleason sum at diagnosis, stage/prognostic group status and the *AKR1C3* rs12529 SNP genotype were considered in this analysis. Therefore data from a total of 376, 202 and 232 NZ, AA and EA patients respectively were used in the current analysis. PC TNM staging data was not considered in this analysis as the NZ cohort had 32% incomplete data. Smoking status was considered as ever smoker if a patient has been a current or a former tobacco smoker. PSA data were stratified into 2ng/ml class intervals between >4-<20ng/ml. Those with the PSA level <4ng/ml and >20ng/ml were pooled into two separate class intervals respectively. PC records were stratified based on the PSA level and further grouped by the tobacco smoking status. US data were separated between AA and EA groups. Data were further stratified based on a dominant model where the *AKR1C3* rs12529 CC and CG+GG were considered as two groups.

### Data analysis

Normally distributed continuous variable age was analysed using the one way ANOVA followed by Holm-Sidak method for follow-up multiple comparison testing. Non-normally distributed continuous variable PSA was analysed using the Kruskal-Wallis One Way ANOVA on ranks followed by Dunn’s method for follow-up multiple comparison testing. Categorical variables were analysed using the Chi Square test.

The percentage of high-risk PCs as a fraction of all cancers was calculated for each PSA class interval and the cumulative % high-risk cancers against all PCs (outcome variables) were recorded under increasing PSA intervals (exposure factor). Similarly, high-risk PCs at each PSA interval as a fraction of total high-risk PCs were also calculated and the cumulative fractions of high-risk PCs against all high-risk PCs were recorded under increasing PSA intervals. These cumulative distributions were further stratified based on tobacco smoking status and the *AKR1C3* rs12529 CC and CG+GG combined genotypes. Cumulative % frequencies between different groups were compared using the Kolmogorov-Smirnov (KS) test [27] assuming the null hypothesis that both groups were sampled from populations with identical distributions. The level of significance was set at 0.05 and the KS critical D value for two unequal sample sizes was estimated as 1.36*sqrt((m+n)/(m*n)) where m and n were the sample size of tested groups [28]. Where the maximum cumulative distribution function (CDF which is the maximum difference in cumulative frequency estimated between two groups for different class intervals) was higher than the estimated critical D value, the difference was considered significant.

## Results

Patient characteristics of the cohorts are summarised in Table 1. The NZ PC cohort has been diagnosed at a significantly (p<0.05) higher mean age (66.67± (8.08) y) compared to both the AA (62.65±8.17y) and EA (64.83+8.56y) groups from US. Tobacco smoking status was not different between the three groups. The median PSA level at diagnosis was the highest in the NZ cohort (8.90ng/ml) and was significantly (p<0.05) different to both AA (6.86ng/ml) and EA (5.80ng/ml) groups. The median in Gleason sum was significantly (p<0.05) different between NZ and EA (7 and 6 respectively) groups. There was no significant difference between stage/prognostic grouping between NZ, AA and EA groups.

**Table 1:** Patient characteristics compared between the study cohorts.

		NZ	AA	EA	P value
Age at diagnosis		66.67	62.65	64.83	NZ vs AA
Mean(SD)		(8.08)	(8.17)	(8.56)	P<0.001
[number]		[376]	[202]	[232]	EA vs AA
					P = 0.012
					NZ vs EA
					P = 0.008
Tobacco smoking	Ever smoker	242(64.4)	136(67.3)	145(64.4)	Chi Square
status					statistic = 1.1127
Number(%)	Never smoker	134(35.6)	66(32.7)	87(35.6)	P = 0.573
PSA at diagnosis		8.90(5.82,15.25)	6.86(4.97,13.41)	5.80(4.70,8.45)	NZ vs AA
					P = 0.006
Median					EA vs AA
					P = 0.002
(25% and 75%)					NZ vs EA
					P<0.001
Gleason sum		7 (6,7)	7 (6,7)	6 (6,7)	NZ vs AA
					P<0.001
Median					NZ vs EA
(25% and 75%)					P <0.001
*Stage/prognostic	<IIB	168 (44.7)	65 (32.2)	102 (44)	NZ vs AA
group					P>0.05
Number (%)	≥IIB	208 (55.3)	137 (67.8)	130 (56.0)	EA vs AA
					P>0.05
					NZ vs EA
					P>0.05

NZ = New Zealanders, AA = African Americans, EA = European Americans

*NZ group had 32% missing data for the TNM staging. In the current analysis high-risk PC was stratified based on a cancer stage of ≥T2C (if available) or PSA≥20ng/ml or a Gleason grade of ≥8 using a recently reported criterion [20, 21]. This high-risk PC stratification fitted well with a cut-off at stage/prognostic group IIB defined by the AJCC 7^th^ edition.

**Fig 1:**
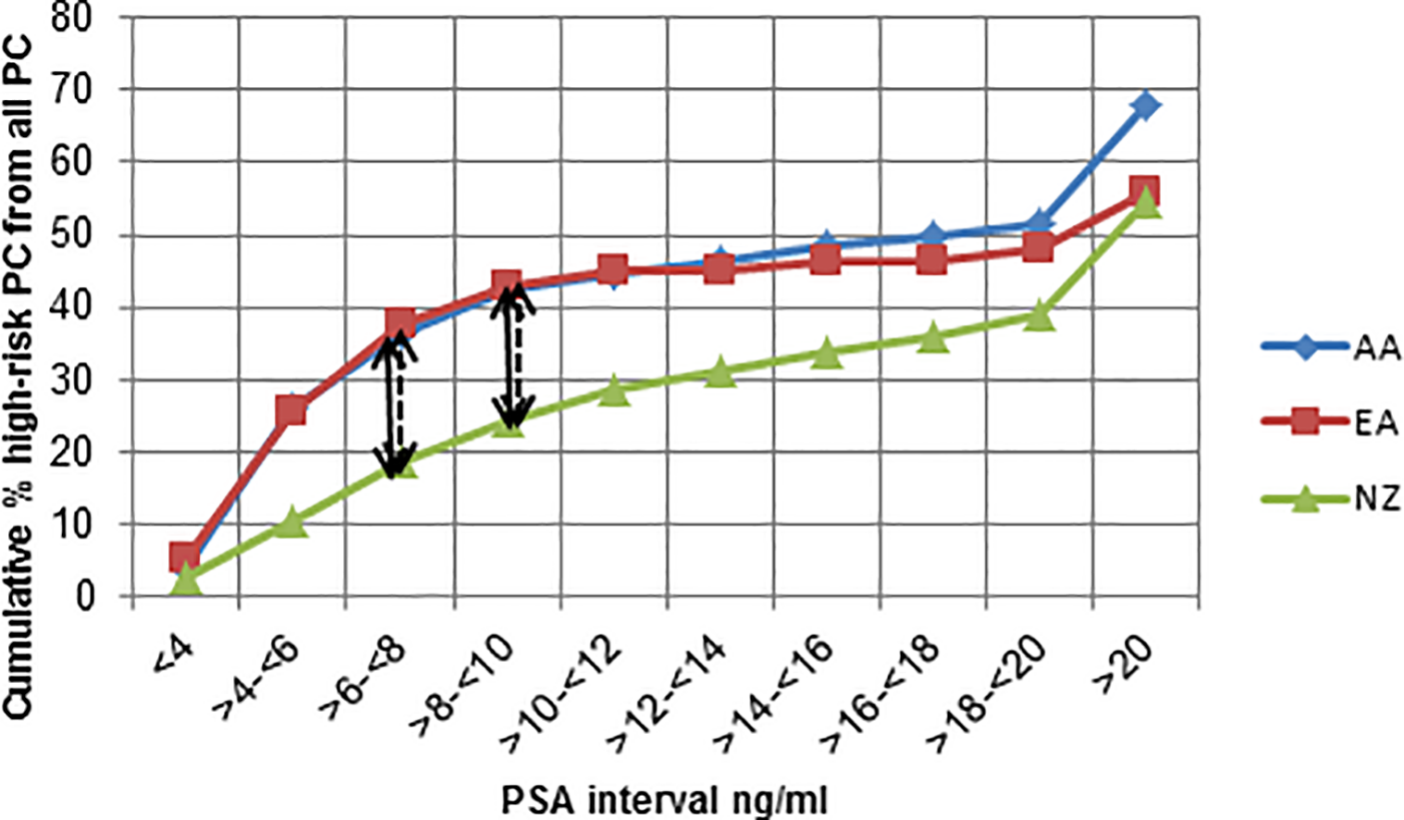
Cumulative % high-risk PC as a fraction of all PCs by PSA groups. (NZ = New Zealanders, AA = African Americans, EA = European Americans. Points of significant differences (p<0.05) are shown according to the Kolmogorov-Smirnov test results. Full double headed arrow = maximum difference between EA and NZ. Dashed double headed arrow = maximum difference between AA and NZ).

High-risk PC constituted 55.3%, 67.8% and 56.0% of all PCs in NZ, AA and EA groups respectively. There is a significant variation in the cumulative % high-risk PCs detected as a fraction of all PCs between EA vs NZ and AA vs NZ at particular PSA intervals. The maximum CDF for high-risk PC detected as a fraction of all PCs between EA and NZ and AA and NZ groups were 0.19 and 0.18 respectively while the critical D value was 0.15 for both comparisons (p<0.05) (Fig 1). These differences occurred at PSA intervals of >6 to ≤8 and >8 to ≤10.

**Fig 2:**
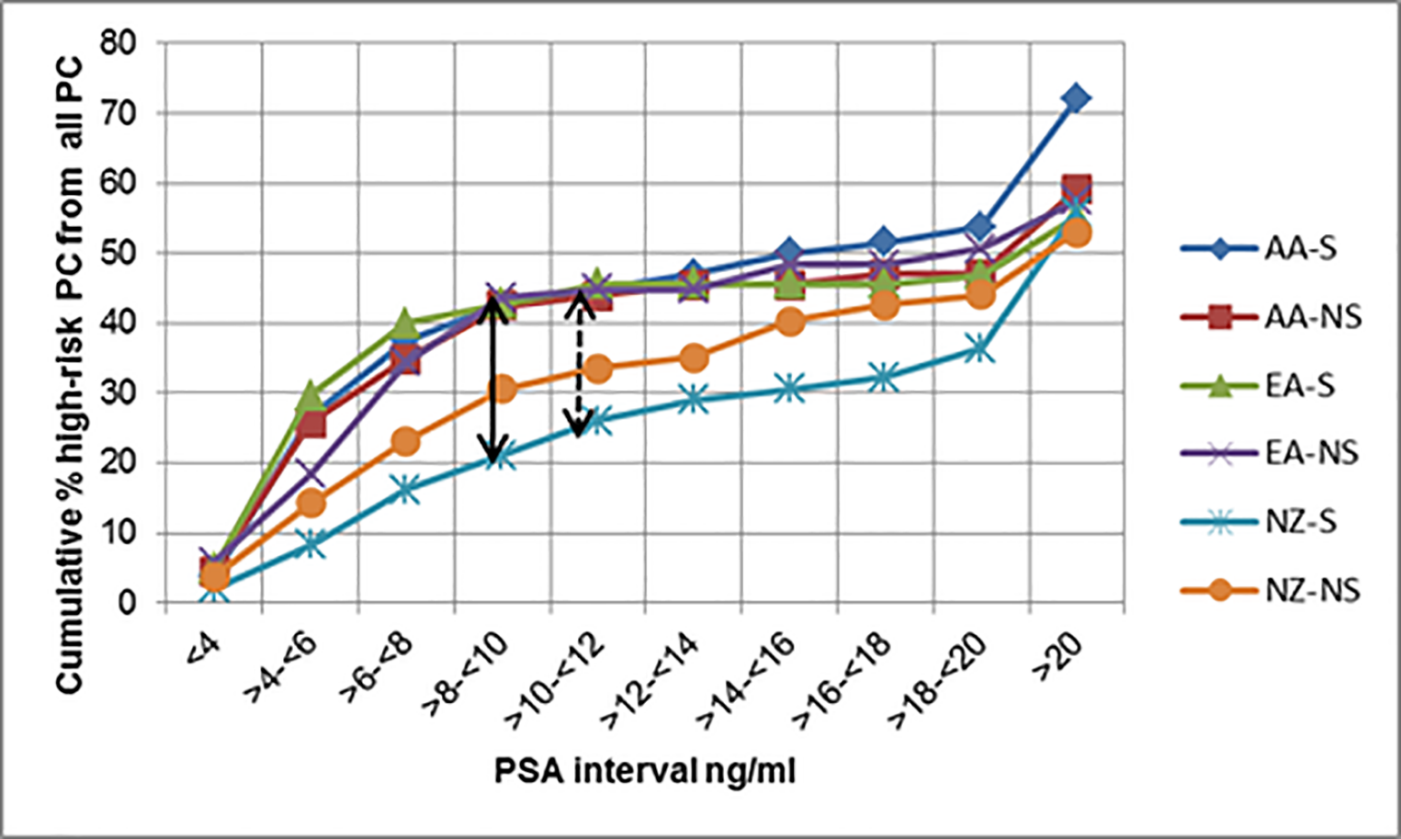
Cumulative % high-risk PC as a fraction of all PCs by PSA groups separated by smoking status. (NZ = New Zealanders, AA = African Americans, EA = European Americans. S = ever smokers NS = never smokers. (Points of significant differences (p<0.05) are shown according to the Kolmogorov-Smirnov test results. Full double headed arrow = maximum difference between EA ever smokers and NZ ever smokers. Dashed double headed arrow = maximum difference between AA ever smokers and NZ ever smokers).

Regardless of ethnic and tobacco smoking variability, an overlapping trend in the cumulative % high-risk PC detection from all PCs with increasing PSA intervals were recorded for both US groups (Fig 2). However, among ever smokers, AA and EA groups showed a higher trend in cumulative % high-risk PC detection from all PCs compared to those from the NZ cohort. The maximum CDF values between EA vs NZ and AA vs NZ were 0.24 and 0.22 respectively while the critical D values were 0.19 and 0.18 respectively for these ever smokers. These differences too occurred at PSA intervals of both >6 to ≤8 and >8 to ≤10ng/ml. However, the difference between these trends between EA/AA groups and NZ cohort remain non-significant among never smokers.

**Fig 3:**
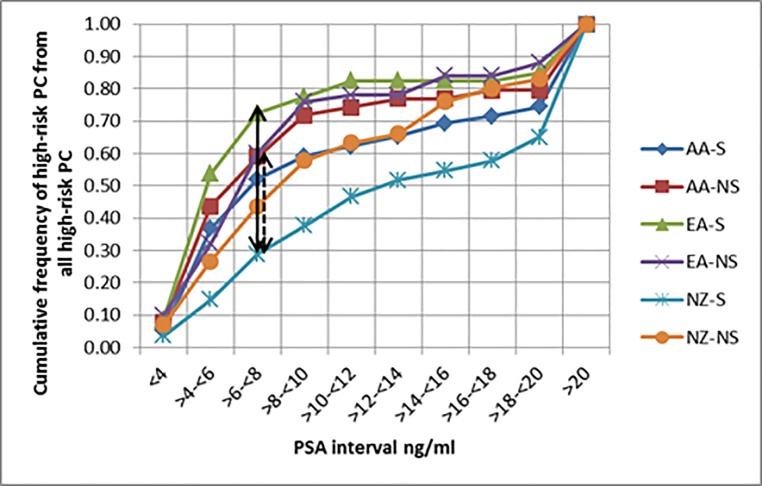
Cumulative frequency of high-risk PC as a fraction of all high-risk PCs by PSA groups in study cohorts. (NZ = New Zealanders, AA = African Americans, EA = European Americans S = ever smokers NS = never smokers. Points of significant differences (p<0.05) are shown according to the Kolmogorov-Smirnov test results. Full double headed arrow = maximum difference between EA and NZ. Dashed double headed arrow = maximum difference between AA and NZ).

The trend in cumulative frequency of high-risk PC from all high-risk PCs were also considered between ever and never smokers of all cohorts. NZ ever smokers showed the lowest trend line (Fig 3). Among NZ ever smokers, 50% of high-risk PCs are detected around a PSA interval of >12- ≤14ng/ml, while for both US groups 50% of all high-risk PCs are detected before reaching the >6-≤8ng/ml PSA interval regardless of tobacco smoking. There is a significant variation in the cumulative high-risk PCs detected as a fraction of all high-risk PCs between US and NZ groups. The maximum CDF for high-risk PC detected as a fraction of all high-risk PCs were 0.44 and 0.23 respectively between EA and NZ and AA and NZ ever smokers respectively while the critical D values for the two comparisons were at 0.19 and 0.18 respectively. This was recorded at a PSA interval of >6-≤8ng/ml. There was a marginal difference in maximum CDF between high-risk PC detected as a fraction of all high-risk PCs between ever smokers of EA and AA groups at the same PSA interval with both the maximum CDF and the critical D value being at 0.20. Among never smokers, no difference in the cumulative high-risk PCs detected as a fraction of all high-risk PCs were observed between study groups.

**Fig 4:**
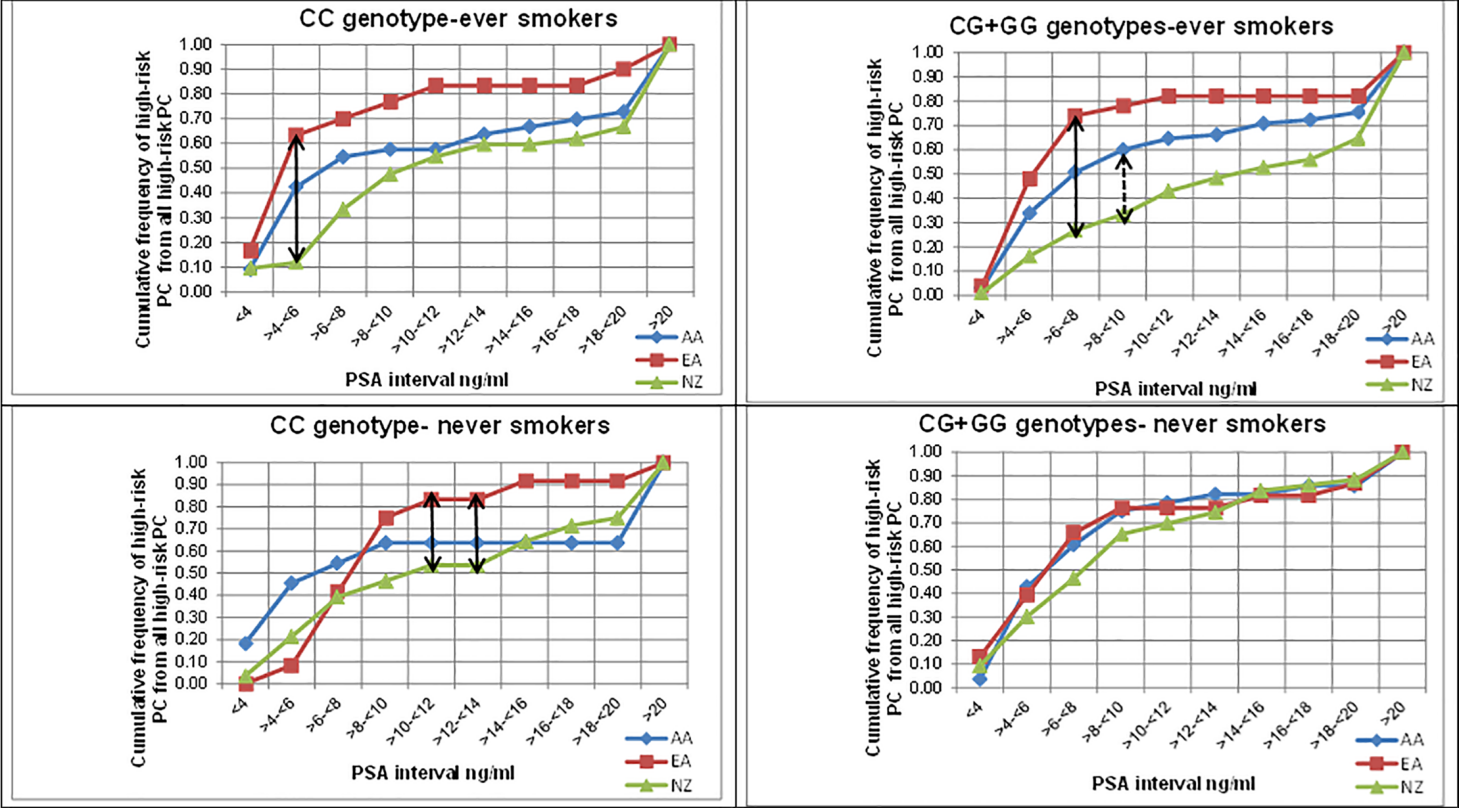
Cumulative frequency of high-risk PCs as a fraction of all high-risk PCs by PSA groups, genotype and tobacco smoking status. (AA = African American EA = Caucasian American NZ = New Zealanders S = ever smokers NS = never smokers. Points of significant differences (p<0.05) are shown according to the Kolmogorov-Smirnov test results. Full double headed arrow = maximum difference between EA and NZ. Dashed double headed arrow = maximum difference between AA and NZ).

Genotype data on the *AKR1C3* rs12529 SNP indicates that all three groups have similar genotype and allele frequencies (Table 2). Table 3 presents high-risk PC data stratified based on the above polymorphism between ever and never smokers in these three groups. The highest percentage of high-risk PC as a percentage of all PC were recorded for ever smoker AA men with the *AKR1C3* rs12529 CC genotype (84.6%) while the lowest was recorded for never smoker NZ men with the CG+GG genotypes (47.2%). The variations in cumulative high-risk PCs as a fraction of all high-risk PCs among ever and never-smokers are shown in Fig 4 for the three groups separated by the genotype. There is a significant variation in the CDF for high-risk PCs detected as a fraction of all high-risk PCs between the US and NZ ever smokers regardless of the genotype variation (Table 4). The maximum CDF values between the EA and NZ cohort were 0.51 and 0.47 for the CC and CG+GG genotypes respectively compared to the critical D values of 0.33 and 0.24 respectively among ever smokers. Among the AA and NZ cohort with CG+GG genotypes, the maximum CDF of 0.27 was higher compared to the critical D value of 0.22 among ever smokers. The maximum CDFs between the EA and NZ cohort with CC genotype and ever smokers are recorded at a PSA interval of >4.0-≤6.0ng/ml. For those with CG+GG genotypes the maximum CDFs are recorded at a PSA intervals of >6.0-≤8.0ng/ml between ever smokers from the EA and NZ cohort (Fig 4 and Table 4). 50% of high-risk PCs among ever smokers carrying CG+GG genotypes in NZ cohort was recorded when the PSA interval has reached around 14ng/ml, while such detection among the EA and AA groups were recorded at PSA intervals of >4-≤6ng/ml and >6-≤8ng/ml respectively (Fig 4). Among those carrying the CC genotype and who were ever smokers, 50% of high-risk PCs are detected around a PSA level of 10ng/ml for the NZ cohort while for the EA and AA groups, this occurs at PSA intervals of >4-≤6ng/ml and >6-≤8ng/ml respectively. The ever smokers of the EA and NZ cohort carrying the *AKR1C3* rs12529 CC genotype show a trend of getting 50% high-risk PCs detected at an earlier PSA interval than that of the never smokers. Never smokers from NZ, AA and EA groups with the *AKR1C3* rs12529 CG+GG genotypes showed a similar variation pattern in % high-risk PC detections with increasing PSA intervals.

**Table 2:** Genotype and allele frequencies recorded between study cohorts.

Genotype numbers and % frequencies					% allele frequency	
	CC	CG	GG	CG+GG	C	G
NZ	119 (31.65)	166 (44.15)	91 (24.20)	257 (68.35)	53.72	46.28
AA	58 (28.71)	105 (51.98)	39 (19.31)	144 (71.29)	54.70	45.30
EA	69 (29.74)	115 (49.57)	48 (20.69)	163 (70.26)	54.53	45.47

NZ = New Zealanders, AA = African Americans, EA = European Americans.

**Table 3:** High-risk PC statistics against genotype, smoking status and PSA interval among study groups.

	NZ				AA				EA			
	ever-smokers		never-smokers		ever-smokers		never-smokers		ever-smokers		never-smokers	
	*AKR1C3* rs12529 genotype											
	CC	CG+GG	CC	CG+GG	CC	CG+GG	CC	CG+GG	CC	CG+GG	CC	CG+GG
numbers with high-risk PC (% from total high-risk PC)												
PSA classification												
<4	4(10)	1(1)	1(4)	4(9)	3(9)	2(3)	2(18)	1(4)	5(17)	2(4)	0	5(13)
>4-<6	1(2)	14(15)	5(18)	9(21)	11(33)	20(31)	3(27)	11(39)	14(47)	22(44)	1(8)	10(26)
>6-<8	9(21)	10(11)	5(18)	7(16)	4(12)	11(17)	1(9)	5(18)	2(7)	13(26)	4(33)	10(26)
>8-<10	6(14)	6(6)	2(7)	8(19)	1(3)	6(9)	1(9)	4(14)	2(7)	2(4)	4(33)	4(11)
>10-<12	3(7)	9(10)	2(7)	2(5)	0	3(5)	0	1(4)	2(7)	2(4)	1(8)	0
>12-<14	2(5)	6(6)	0	2(5)	2(6)	1(2)	0	1(4)	0	0	0	0
>14-<16	0	4(4)	3(11)	4(9)	1(3)	3(5)	0	0	0	0	1(8)	2(5)
>16-<18	1(2)	4(4)	2(7)	1(2)	1(3)	1(2)	0	1(4)	0	0	0	0
>18-<20	2(5)	8(9)	1(4)	1(2)	1(3)	2(3)	0	0	2(7)	0	0	2(5)
>20	14(33)	35(35)	7(25)	5(12)	9(27)	16(20)	4(36)	4(14)	3(10)	9(18)	1(8)	5(13)
Total high-risk PCs	42	95	28	43	33	65	11	28	30	50	12	38
Total PC	76	166	43	91	39	97	19	47	51	94	18	69
% of high-risk PC from the total PC	55.3	57.2	65.1	47.2	84.6	67.0	57.9	59.6	58.8	53.2	66.7	55.7

NZ = New Zealanders, AA = African Americans, EA = European Americans

**Table 4:** Genetic and ethnic stratification of high-risk PCs as fractions of all high-risk PCs in each PSA interval.

Ever-Smokers - Kolmogorov-Smirnov CDF test statistic						
	*AKR1C3* CC genotype			*AKR1C3* CG+GG genotypes		
PSA class	EA- AA	EA- NZ	AA- NZ	EA- AA	EA- NZ	AA- NZ
<4	0.08	0.07	0.00	0.01	0.03	0.02
>4-≤6	0.21	**0.51**	0.31	0.14	0.32	0.18
>6-≤8	0.15	0.37	0.21	0.23	**0.47**	0.24
>8-≤10	0.19	0.29	0.10	0.18	0.45	**0.27**
>10-≤12	**0.26**	0.29	0.03	0.17	0.39	0.22
>12-≤14	0.20	0.24	0.04	0.16	0.34	0.18
>14-≤16	0.17	0.24	0.07	0.11	0.29	0.18
>16-≤18	0.14	0.21	0.08	0.10	0.26	0.16
>18-≤20	0.17	0.23	0.06	0.07	0.17	0.11
>20	0.00	0.00	0.00	0.00	0.00	0.00
Critical D (EA vs AA)		0.34		0.26		
Critical D (EA vs NZ)		0.33		0.24		
Critical D (AA vs NZ)		0.32		0.22		
Never Smokers- Kolmogorov-Smirnov test statistic						
PSA class	EA- AA	EA- NZ	AA- NZ	EA- AA	EA- NZ	AA- NZ
<4	-0.18	-0.04	0.15	0.10	0.04	-0.06
>4-<6	-0.37	-0.13	0.24	-0.03	0.09	0.13
>6-<8	-0.13	0.02	0.15	0.05	0.19	0.14
>8-<10	0.11	0.29	0.17	0.01	0.11	0.10
>10-<12	0.20	**0.30**	0.10	-0.02	0.07	0.09
>12-<14	0.20	**0.30**	0.10	-0.06	0.02	0.08
>14-<16	0.28	0.27	-0.01	-0.01	-0.02	-0.02
>16-<18	0.28	0.20	-0.08	-0.04	-0.04	0.00
>18-<20	0.28	0.17	-0.11	0.01	-0.02	-0.03
>20	0.00	0.00	0.00	0.00	0.00	0.00
Critical D (EA vs AA)		0.57		0.34		
Critical D (EA vs NZ)		0.16		0.30		
Critical D (AA vs NZ)		0.48		0.33		

NZ = New Zealanders, AA = African Americans, EA = European Americans.

Bold = points of significant differences

## Discussion

In New Zealand (NZ), prostate cancer (PC) is the most commonly registered male cancer and the third most common cause of cancer deaths [29]. According to this report from the NZ Ministry of Health, PC registration rates of 99.1 and 82.1 per 100,000 age-standardised to the WHO World Standard population were recorded for non-Māori and Māori populations respectively in 2012 [29]. In the United States (US), this is the most common cancer in men after skin cancer and the second leading cause of cancer death [30]. According to Etzioni et al PC mortality in US declined by 35% since its peak in 1990s [31]. These authors also note that the incidence of late-stage disease declined by 75% during this period. A fixed-cohort simulation model on PC progression and screening has indicated that 80% of the observed decline in late-stage disease from 1990s to mid-2000s is due to PSA screening [32]. These authors believe that the rest of the decline is due to PC awareness and advances in treatment methods. According to US PC incidence statistics a rate of 121.9 and 203.5 per 100,000 and age-adjusted to the 2000 US Standard Population were recorded for the European American (EA) and African American (AA) populations between the period 2009–2013 [33].

Our analysis shows a disparity of mean age and median PSA at PC detection between the NZ and US cohorts. The median Gleason sum for NZ cohort was also significantly higher than the EA group. Pokorny et al records results of a retrospective audit of all men undergone prostate biopsy at the Urology Department, Auckland, Hospital, NZ in 2005–2006 [34]. The median age and the PSA level at PC diagnosis in our current NZ cohort seem to have improved compared to the results from the said audit [34]. The median age at PC diagnosis was 67.5y (current analysis) compared to 69y and 68y for NZ Europeans and NZ Maori/Pacific PC patients respectively recorded in the 2006 audit. Similarly the median PSA at diagnosis was 8.9ng/ml (current analysis) compared to 12.6ng/ml and 13.3ng/ml NZ Europeans and NZ Maori/Pacific PC patients respectively in the said audit. However, these improvements have not reached the levels reported in the current US cohort.

Screening of men in the Prostate Testing for Cancer and Treatment (ProtecT) trial have excluded 16% of men with high-risk disease (categorised as men identified with locally advanced (clinical stage T3–4) or metastatic (N1 or M1) PC, as well as those with PSA >20 ng/ml) [35, 36]. Proportions of PC with similar characteristics in our study cohorts were 15.9 for EA, 20.3% for AA and 31.5% for NZ (results not shown). This means that high-risk PC detected in the ProtecT trial as well as the EA group from the NCI cohort are typical for screen-detected high-risk PC as described before [36, 37]. However, the proportion of high-risk PC in the NZ cohort was almost double that of the general trend in screened detected PC. A general delay in the diagnosis of high-risk PC is evident in the NZ cohort which gets aggravated among ever smokers. This situation is further compounded with the genetic interaction. The never smoker patients in all three study groups with the *AKR1C3* rs12529 CG and GG genotypes show similar trends for the cumulative % high-risk disease recordings with increasing PSA intervals. For those with the same genotypes, but who are ever smokers, % high-risk PC recording is severely impacted in the NZ cohort compared to the two US groups. Among the reasons for this difference could be NZ men with a lifestyle that includes tobacco smoking having lower serum PSA levels associated with the *AKR1C3* rs12529 G allele as reported by us before [13] and escaping early cancer detection through current PSA cut-off thresholds. High-risk PC recording in the NZ ever smokers carrying the *AKR1C3* rs12529 CC genotype is also impacted compared to the two US groups. However, the ever smokers of the EA and NZ cohorts carrying the *AKR1C3* rs12529 CC genotype seem to get relatively early diagnosis of high-risk PC at relatively lower PSA levels compared to the never smokers from these cohorts. This again could be due to the *AKR1C3* rs12529 CC genotype showing a positive association with the PSA levels as shown by us before [13] and their high-risk PCs getting captured at PSA testing with current cut-off thresholds. Our studies have previously recorded that the *AKR1C3* rs12529 C allele carries a risk of aggressive PC when interacting with lifestyle factors including tobacco smoking in a NZ cohort [38]. Meanwhile, concurrent use of tobacco and alcohol, and the former leading to the latter has been reviewed by Cross et al [39]. Therefore, the delayed diagnosis we see with tobacco smokers could have an underlying influence of alcohol consumption effect as well.

According to predictive modelling by Gulati et al, discontinuing PSA screening in the US will generate many cancer deaths that could have been avoided under a screening strategy [40]. These authors point out that if screening is carried out with a cut-off age of 70 y, 50% of avoidable cancer deaths could be prevented while minimising over-diagnosis. According to Fleshner et al [5] an increase in higher grade tumors and higher staging in PCs at detection is recorded subsequent to the 2012 USPSTF PSA recommendations. According to these authors during the first few years of the UPSPSTF recommendations on PSA screening, there was a decline in the rates of PSA screening, prostate biopsies and overall PC incidence in the US. However, the authors also note a subsequent increase in higher grade tumors and higher staging in PCs at detection.

According to an analysis by Hutchinson et al [41], the proportion of referrals for further investigation of men following a PSA exam has dropped down from around 0.045–0.055 (during 2010–2012) to 0.03 in 2015 at a US centre. According to statistics given in Obertova et al [42], a comparative figure for the Midlands region in NZ was 0.019 and 0.008 referrals per PSA exam respectively for Māori and non-Māori, both of which are way below the estimates made by Hutchison et al including recordings for the post- UPSTF PSA recommendation period. According to Hutchinson and co-workers [41], the average level of PSA at patient referral for further examination has been rising from 2.56ng/ml in 2012 to 3.84ng/ml during the period beyond 2015. However, the median level of PSA at referral for further assessment in NZ in 2010 were 3.2ng/ml (40-49y), 5.9ng/ml (50-59y), 7.5ng/ml (60-69y), 9.9ng/ml (70-79y) and 16.6ng/ml (above 80y) [8]. The pre-biopsy PSA levels evaluated in a US cohort at one and three years since the UPSTF PSA recommendation have been 7.0 and 8.1ng/ml respectively showing a gradual increase with time [43] and getting closer to the median PSA level at diagnosis of the NZ cohort evaluated in our study. Gejerman et al records a shift of median Gleason sum from 6 to 7 between the periods 2011 and 2014 [44]. The Gleason variation between these two periods is comparable to the Gleason variation between the EA and NZ cohorts reported in our study.

According to Gaylis et al [43], if this trend of diagnosis at higher PSA continues in the US, it may end up in an era similar to that of the pre-PSA times. This will invariably create a situation where PC gets detected at locally advanced or metastatic disease where androgen deprivation therapy and palliative care become the treatment options [45]. This author calls for abandoning the ‘one size fits all’ recommendation on PSA screening and to look out for more personalised approaches for screening for maximum benefits while minimizing harms of screening.

Delayed PC diagnosis is a public health burden with increased treatment costs. Studies show a 24–75% increase in treatment costs from low- to high- risk PCs that use various radiation therapy (RT) options, while for surgical options it is 71–79% higher than that of low-risk cancers [46]. As Cooperberg et al records, RT is a more expensive procedure compared to surgery [46]. Androgen deprivation therapy (ADT) is an effective treatment in men with advanced metastatic PC and those with high-risk tumors in combination with radiation therapy (RT) [47]. Meanwhile, ADT comes with adverse events in 50% of patients and can produce additional healthcare costs of 99% for adverse diabetes outcomes to as high as 245% for multiple adverse events and 524% for end stage costs compared to baseline ADT cost [48].

The CONCORD study on global surveillance of cancer survival carried out with statistics from1990-1999 indicates that the relative survival of age standardised PC rate was 91.9% in US while that of Australia was 77·4% [49]. The more recent CONCORD 2 study with statistics between 1995–2009 including that of NZ data indicates that the relative survival of age standardised PC rate among US, NZ and Australia are 93.2%, 88.7% and 88.5% respectively [50]. This indicates improvement of the percentage relative survival of age standardised PC rates during the latter period in both US and Australia, and NZ sharing similar rates with Australia. However, a recent study by Sandiford et al [51] has compared the % of avoidable excess death due to various cancers between NZ and Australia from 2006–2010. This study indicates that 25.7% avoidable excess cancer deaths in NZ are due to PC. There is no literature available on comparison of PC mortality rates between US and NZ using a similar age standardised approach. However, a comparison of mortality rates has been made between Australia and NZ between 2002–2007 [52]. According to these authors, NZ records 9% more PC related mortalities compared to that of Australia. A comparison of mortality rates between US, UK, Canada and Australia has reported that Australia and UK record significantly higher mortality rates compared to US [53]. These indirectly indicate that PC related mortalities are comparatively higher in NZ than in US. Unfortunately the above comparative studies have not stratified data to assess the impacts of tobacco smoking on PC mortality.

High-risk PC recording at higher PSA class intervals in NZ cohort compared to US cohorts could be indicating consequences of low level PC screening as recorded in NZ. This discrepancy was further compounded by tobacco smoking in this NZ cohort. Therefore, it is worth considering men who have been ever smokers for PC screening even if other restrictions are in place with regards to PSA based screening. Delayed diagnosis of high-risk PC in NZ could be among the reasoning behind lower relative survival of age standardised PC rate in NZ compared to that of the US [50]. We have previously shown that the frequency of the *AKR1C3* rs12529 G allele is 14% higher among NZ men of Māori/ Pacific/East Asian origin compared to NZ Europeans [54]. It is also known that Māori men have a higher PC related mortality rate than that of the non-Māori PC patients in NZ [29]. Tobacco smoking rates are also higher in Māori men compared to non-Māori men in NZ [55]. We have also recorded that the *AKR1C3* rs12529 G allele is associated with lower levels of PSA (and a higher level of PSA associated with the C allele) among PC patients when compared to healthy controls when interacting with lifestyle factors [13]. Impact of this SNP variant has not yet been studied separately in Māori men. However, lowering PSA screening thresholds for those carrying the *AKR1C3* rs12529 G allele with a higher tendency of tobacco smoking (such as Māori men) could enable early diagnosis of significant PCs in this stratified group. Similarly, there is a possibility of those carrying the AKR1C3 rs12529 CC genotype to be over-diagnosed and over-treated for less significant PC as they carry higher PSA levels when interacting with lifestyle factors. Therefore it could be beneficial to study this aspect further for establishing genotype and lifestyle based PSA thresholds.

One limitation of the current analysis is recruitment times of the two cohorts being different at the beginning and at the end of completion. The US cohort recruitment was initiated around one year ahead (in 2005) of the NZ cohort and recruitment was completed two years after (in 2015) that of the NZ cohort. There is a possibility that in the period 2014–2015, the US cohort would have been subjected to PSA based screening limitation impacting diagnosis. Another limitation could be the 32% missing data for the TNM staging in the NZ cohort that could have impacted the stage/prognostic grouping. The NZ group having been diagnosed at a significantly late age compared to AA and EA groups also could have compounded the results. Additionally, the NZ cohort was recruited with limitations until August 2010 and that also could have impacted patient catchment. The current analysis looked into the high-risk PC diagnoses variability reported in patients only and therefore cannot be extrapolated to men without known PC.

## Conclusion

Findings from our current analysis indicate that there is delayed diagnosis of those with high-risk PC in NZ, and that the situation is far worse with those that have had a tobacco smoking lifestyle. We also see that delayed diagnosis of high-risk PC among men with tobacco smoking lifestyle gets further impacted by genetics. Several factors including lower levels of PSA screening, delayed referral to specialist care and lower subsequent biopsies on those with elevated PSA levels compared to that of US could be underlying reasons for this discrepancy that require attention from the NZ health authorities.

## Supporting information

S1 Table The Standard Strengthening the Reporting of Genetic Association Studies [STREGA]–an extension of the STrengthening the Reporting of OBservational studies in Epidemiology [STROBE] statement is provided in S1 Table -summary of details related to batch genotyping for the *AKR1C3* rs12529 SNP. (DOCX)

S2 Table All data used in this analysis are given in S2 Table Datafile. (XLSX)

## Abbreviations

AA: African American
AJCC: American Joint Committee on Cancer
CDF: Cumulative distribution function
EA: European American
EDTA: Ethylenediaminetetraacetic acid
ERSPC: European Randomized Study of Screening for Prostate Cancer
KS: Kolmogorov-Smirnov
NCI: National Cancer Institute
NZ: New Zealand
PC: Prostate cancer
PLCO: US Prostate, Lung, Colorectal and Ovarian Cancer screening trial
PCR: Polymerase chain reaction
PSA: Prostate-specific antigen
SNP: Single nucleotide polymorphism
SQRT: Square root
TNM: tumor–node–metastasis
WHO: World Health Organisation
US: United States
USPSTF: United States Preventive Services Task Force
