# Supplementary material for: Influence of lifestyle and genetic variants in the aldo-keto reductase 1C3 rs12529 polymorphism in high-risk prostate cancer detection variability assessed between US and New Zealand cohorts
Source: PLoS One. 2018 Jun 19;13(6):e0199122. doi: 10.1371/journal.pone.0199122 (PMC6007906; doi:10.1371/journal.pone.0199122)
Supplement: S1 Table — (DOCX) [file pone.0199122.s001.docx]

**S1 Table -Summary of details related to batch genotyping for the *AKR1C3* rs12529 SNP**

(Details provided as a requirement of the Standard Strengthening the Reporting of Genetic Association Studies [STREGA]–An Extension of the STrengthening the Reporting of OBservational studies in Epidemiology [STROBE] Statement.)

| **1a. DNA extraction, quantitation and purity checking** | |
| --- | --- |
| Total genomic DNA extraction | *NZ cohort*-  QIAamp DNA Blood Mini Kit (Catalogue # 51106 from Qiagen, Hilden, Germany) according to the manufacturer’s instructions using a fully automated procedure on the QIAcube -230V (Catalogue # 9001293 from Qiagen, Hilden, Germany).  *NCI cohort from US-*  DNeasy blood and tissue kit from Qiagen (Catalogue # ID: 69506, Qiagen Hilden, Germany) following manufacturers’ instructions. |
| DNA quantitation and purity checking and dilution | NanoDrop 1000 v 3.8. ( Thermo Scientific, Wilmington, USA) |
| DNA storage and dilution for genotyping | DNA samples were stored at -20^0^C. All DNA samples were normalised to 10ng µl^-1^. |

| **1b. Genotyping methods** | | |
| --- | --- | --- |
| **Method** | Sequenom’s genotyping iPLEX Assay (Sequenom)* | TaqMan SNP Genotyping (Applied Biosystems)** |
| Primer design | MassArray Assay Design Software v 3.0 was used to design a multiplex SNP genotyping assay that included the AKR1C3 rs12529 | Predesigned by assay on demand (C__8723970_1_from the Applied Biosystems |
| Genotyping assay | According to manufacturer’s specifications (Sequenom, San Diego, CA, USA) | Reactions prepared based on Ferguson et al ^[26]^ |
| Detection method | Matrix-assisted laser  desorption/ionization-time of flight  (MALDI TOF) mass spectrometry.  (Spotting and firing of samples were carried out at AgResearch Ltd, Invermay Agricultural Centre, Puddle Alley, Mosgiel, New Zealand.) | Sequence detection system SDS v 2^.^4 software.  These were carried out at the Auckland Cancer Society Research Centre, University of Auckland, |
| Genotype calling software | iPLEX MassARRAY  Typer v.4^.^0 software |  |

| **1c. Details of batch genotyping** | | | | |
| --- | --- | --- | --- | --- |
| Batch | Operator/method | DNA Storage time in -20^0^C before genotyping | Total number assayed | Total produced results  (% call rate) |
| *All Urology patients (with prostate cancer and with benign urology disease) genotyped in NZ* | | | | |
| NZ 1 | Katja Lange- Sequenom | 2y 2m | 178 | 176 (98^.^9) |
| NZ 2 | Alice Wang- TaqMan | 3y 2m | 313 | 305 (97^.^4) |
| NZ 3 | Alice Wang - TaqMan | 1y 3m | 96 | 96 (100) |
| NZ 4 | Alice Wang - TaqMan | <1y | 45 | 43 (95^.^6) |
| NZ 5 | Alice Wang- TaqMan | <1y | 19 | 19 (100) |
| *Prostate cancer patients from the NCI, US* | | | | |
| US1 | TaqMan | 2y | 504 | 474(94) |

| **1d. Batch genotype and allele numbers** | | | | | | |
| --- | --- | --- | --- | --- | --- | --- |
| Batch | Observed genotype numbers | | | | Observed allele numbers | |
|  | CC | CG | GG | Total | C | G |
| NZ 1 | 53 | 82 | 41 | 176 | 188 | 164 |
| NZ 2 | 107 | 136 | 62 | 305 | 350 | 260 |
| NZ 3 | 32 | 40 | 24 | 96 | 104 | 88 |
| NZ 4 | 12 | 20 | 11 | 43 | 44 | 42 |
| NZ 5 | 6 | 9 | 4 | 19 | 21 | 17 |
| US1 | 144 | 234 | 96 | 474 | 522 | 426 |

| **1e. Observed and expected allele frequencies from each batch** | | | | | |
| --- | --- | --- | --- | --- | --- |
| Batch | Observed allele frequencies | | Expected genotype frequencies | | |
|  | C | G | CC | CG | GG |
| NZ 1 | 0^.^53 | 0^.^47 | 0^.^29 | 0^.^50 | 0^.^22 |
| NZ 2 | 0^.^57 | 0^.^43 | 0^.^33 | 0^.^49 | 0^.^18 |
| NZ 3 | 0^.^54 | 0^.^46 | 0^.^29 | 0^.^50 | 0^.^21 |
| NZ 4 | 0^.^51 | 0^.^49 | 0^.^26 | 0^.^50 | 0^.^24 |
| NZ 5 | 0^.^55 | 0^.^45 | 0^.^31 | 0^.^49 | 0^.^20 |
| US1 | 0.55 | 0.45 | 0.30 | 0.49 | 0.20 |

| **1f. Expected genotype numbers and Hardy-Weinberg equilibrium*** statistics for each batch. (Significance set at p<0.05)** | | | | |
| --- | --- | --- | --- | --- |
| Batch | Expected genotype numbers | | | p-value |
|  | C/C | CG | GG |  |
| NZ 1 | 50^.^20 | 87^.^59 | 38^.^20 | 0^.^40 |
| NZ 2 | 100^.^41 | 149^.^18 | 55^.^41 | 0^.^12 |
| NZ 3 | 28^.^17 | 47^.^67 | 20^.^17 | 0^.^12 |
| NZ 4 | 11^.^26 | 21^.^49 | 10^.^26 | 0^.^65 |
| NZ 5 | 5^.^80 | 9^.^39 | 3^.^80 | 0^.^85 |
| US1 | 143.72 | 234.57 | 95.72 | 1 |

| **1g. Availability of the *AKR1C3 rs12529* genotype data matched to clinical data** | | | |
| --- | --- | --- | --- |
| *AKR1C3 rs12529* genotypes | CC | CG | GG |
| NZ | 119 | 166 | 91 |
| AA | 58 | 105 | 39 |
| EA | 69 | 115 | 48 |

DNA extraction from NZ cohort was carried out using the QIAamp DNA Blood Mini Kit (Catalogue # 51106 from Qiagen, Hilden, Germany). A fully automated procedure on the QIAcube -230V (Catalogue # 9001293 from Qiagen, Hilden, Germany) was followed according to the manufacturer’s recommendations. Extracted DNA was checked for the quality and quantity using the NanoDrop 1000 v 3.8. (Thermo Scientific, Wilmington, USA), and stored at -20^0^C. All DNA samples were normalised to 10ng µl^-1^. DNA extraction from the US cohort was from buffy coats prepared from heparinised blood as follows. Blood was spun at 850g for 10min at 4^0^C and plasma was aspirated. Buffy coat was removed from the remaining red blood cell pellet and washed once in phosphate buffered saline pH 7.4. DNA was extracted from buffy coats using the DNeasy blood and tissue kit from Qiagen (Catalogue # ID: 69506 Qiagen Hilden, Germany)

*The first batch (NZ1) of genotyping was carried out using Sequenom MassArray and iPlex system ( Jurinke et al ^[23]^ and Storm et al ^[24]^) according to manufacturer’s specifications (Sequenom, San Diego, CA, USA). The current *AKR1C3* rs12529 SNP was analysed as part of a multiplexed SNP genotype assay to evaluate association of androgen pathway related SNPs for the risk of PC. The MassArray Assay Design Software v 3^.^0 was used to design a multiplex SNP genotyping assay. The subsequent procedures including assay optimisation, PCR reactions, cleaning of the amplified product, allele discrimination reactions, spotting onto SpectroCHIP microarray, and MALDI-TOF mass spectrometry are described elsewhere (Morgan et al ^[25]^ and Ferguson et al ^[26]^). A total of 8 no-template controls (NTC), 10 HapMap controls (Centre d’Etude du polymorphism Human (CEPH) samples from Utah residents with ancestry from northern and western Europe), test samples, and 8 duplicate samples were assayed in one plate. Spotting and firing of samples were carried out at AgResearch Ltd, Invermay Agricultural Centre, Puddle Alley, Mosgiel, New Zealand. Data analysis was carried out using the iPLEX MassARRAY Typer v.4^.^0 software. Calls on HapMap control samples in this multiplex assay were 99^.^4% identical with data given in HapMap Genome Browser release #28 (<http://hapmap.ncbi.nlm.nih.gov>).

**The rest of NZ patients were genotyped using the TaqMan® SNP Genotyping Assay using allele-specific, dual-labelled hybridization probes [predesigned by assay on demand (C__8723970_1_from the Applied Biosystems]. The reaction preparation is described in Ferguson *et al*.^1^ At assay optimisation a total of 4 NTC and 20 HapMap CEPH controls were assayed. Thereafter, each batch was assayed with 7-8 NTC, 6-8 HapMap CEPH controls, and 3-4 duplicate samples except in the analysis of the last batch where only two duplicate samples were used. The assay was run on the Applied Biosystems 7900HT Fast Real-Time PCR System at the Faculty of Medical and Health Sciences, the University of Auckland. The PCR conditions for the assay were: 10 min 95 °C enzyme activation followed by 40 cycles at 92 °C for 15 s and 60 °C for 1 min (annealing/extension). The allelic discrimination results were determined after amplification by performing an endpoint read using sequence detection system SDS v 2^.^4 software. Calls on HapMap control samples were 100% identical with data given in HapMap Genome Browser release #28 (<http://hapmap.ncbi.nlm.nih.gov>). Genotyping call rate for test samples varied between 95^.^6-100%. Genotype data collected with the above procedures from our previous studies Karunasinghe et al^[13]^ as well as updated genotype data collections were matched to 376 patients from the current study. (S2 Table Datafile). For the US cohort, the same procedure was followed with the Applied Biosystems 7500HT Fast Real-Time PCR System

***Genotype data for the all batches were assessed for the Hardy-Weinberg Equilibrium using a two allele system applying the binomial theorem.
